# Small Pox in Scotland

**Published:** 1863-04

**Authors:** 


Small Pox in Scotland.—During the past year the small pox was very prevalent at Edinburgh and Leith. In the Report of the Registrar-General, it is stated that the disease was carried from Leith to several parts of Scotland, which raises the important question whether some legislative enactment is not necessary to prevent persons laboring under epidemic disease from travelling by railways, steamboats, or other public conveyances.—*London Lancet.*

